# Evidence of feedback regulation of C-type natriuretic peptide during Vosoritide therapy in Achondroplasia

**DOI:** 10.1038/s41598-021-03593-1

**Published:** 2021-12-20

**Authors:** Timothy C. R. Prickett, Eric A. Espiner, Melita Irving, Carlos Bacino, John A. Phillips, Ravi Savarirayan, Jonathan R. S. Day, Elena Fisheleva, Kevin Larimore, Ming Liang Chan, George S. Jeha

**Affiliations:** 1 Department of Medicine, University of Otago, Christchurch, PO Box 4345, Christchurch, 8140 New Zealand; 2 Guy’s and St. Thomas’ NHS Foundation Trust, Evelina Children’s Hospital, London, UK; 3 Baylor College of Medicine, Houston, TX USA; 4 Vanderbilt University Medical Center, Nashville, TN USA; 5 Murdoch Children’s Research Institute, Royal Children’s Hospital Victoria, University of Melbourne, Parkville, VIC Australia; 6 BioMarin Pharmaceutical, Novato, CA USA

**Keywords:** Bone development, Peptides, Growth disorders

## Abstract

Evidence from genetic disorders of CNP signalling suggests that plasma concentrations of CNP are subject to feedback regulation. In subjects with Achondroplasia (Ach), CNP intracellular activity is suppressed and plasma concentrations are raised but the therapeutic impact of exogenous CNP agonists on endogenous CNP is unknown. In this exploratory dose finding and extension study of 28 Ach children receiving Vosoritide over a 5 year period of treatment, endogenous CNP production was assessed using measurements of plasma aminoterminal proCNP (NTproCNP) adjusted for age and sex and normalised as standard deviation score (SDS), and then related to skeletal growth. Before treatment NTproCNP SDS was raised. Within the first 3 months of accelerating growth, levels were significantly reduced. Across the 5 years of sustained growth, levels varied widely and were markedly increased in some subjects during adolescence. Plasma NTproCNP was suppressed at 4 h post-injection in proportion to the prevailing level of hormone resistance as reflected by SDS before injection. We conclude CNP remains subject to regulation during growth promoting doses of Vosoritide. Fall in CNP during accelerating growth is consistent with an indirect feedback whereas the fall at 4 h is likely to be a direct effect from removal of intra cellular CNP resistance.

## Introduction

C-type natriuretic peptide (CNP) is a paracrine growth factor widely expressed across numerous tissues^1^ with diverse functions including regulation of endochondral bone growth, blood flow and pressure in the microcirculation, anti-inflammatory actions, gamete maturation and neurogenesis and connectivity^2^. The best defined of these factors in humans is the crucial role of the hormone in skeletal growth in driving growth plate expansion. Studies in experimental animals show conclusively that it is the local production of CNP acting via its specific receptor NPR2 within growth plate tissues that determines physiological endochondral bone growth^3^. Study of the dynamic role of CNP in the growth of children is challenging due to the rapid clearance of CNP and very low concentrations in plasma. However, an inactive portion of the synthesised product in tissues (proCNP)—aminoterminal proCNP (NTproCNP)—is not subject to clearance or rapid degradation. Its level in plasma reflects variations in linear growth velocity throughout growth in both children and experimental animals^4^. Notably in subjects with genetic disorders of skeletal growth affecting CNP pathway activity plasma NTproCNP concentrations are raised where intra cellular CNP pathway activity is reduced^5,6^ and are reduced where intra cellular activity is enhanced^7–9^. In achondroplasia, the normal reciprocal antagonism^10^ between FGFR3 pathway activity (inhibitory to endochondral bone growth) and CNP signalling (stimulating bone growth) is overridden by a gain of function mutation in FGR3^11^, reducing intracellular CNP activity, and is associated with modest elevations in concentrations of CNP products in plasma^6^. Collectively these findings suggest that CNP production is subject to feedback regulation. Nothing is known of the dynamics or significance of feedback regulation of CNP during periods of active long bone growth. Further, it is unclear whether such feedback is direct or time-dependent on inter-cellular growth responses of skeletal tissues (indirect feedback). Direct feedback results from actions of the cell’s own product on CNP production whereas indirect feedback involves a longer loop mediated by cells other than those secreting the peptide. However in a recent report addressing these important issues in rodent pups, exogenous CNP administered at high concentrations continuously for 3 days inhibited CNP gene expression but only in tissues containing growth plates^12^. Since gene expression was unchanged at 4 h or 24 h after exposure to high doses in vitro or ex vivo, the authors concluded that autoregulation—if present during normal growth—was likely to be indirect and not dependent on gene transcription. Here we report novel observations in real time on the impact of an exogenous CNP analogue (Vosoritide) on endogenous CNP production in children with Achondroplasia (Ach) during a 5-year period of daily treatment^13^. Our hypotheses were that: (1) daily dosing of Vosoritide—depending on dose and duration—inhibits endogenous CNP secretion during phases of increasing growth velocity (indirect feedback) and (2) endogenous CNP will be unaffected by Vosoritide administered 4 h previously (direct feedback).

## Results

### Baseline NTproCNP is elevated relative to the general population in subjects with Achondroplasia

Baseline values of plasma NTproCNP at screening were raised (mean SDS 0.66 ± 0.17, P < 0.001) despite significantly lower AGV (mean 3.9 ± 0.3 cm/year) when compared to general population children^14^ of this age group. Relevant baseline data of the four cohorts relating to age, plasma NTproCNP and AGV at screening^13^ are shown in Table 1 along with the increment in AGV after 6 months of therapy. NTproCNP SDS was lower in Cohort 1, and age was lower in Cohort 4. Cohorts 3 and 4 receiving higher doses of Vosoritide (15 and 30 µg/kg/day respectively) exhibited significant and similar increase in AGV as assessed at 6 months (P < 0.05 for both).

**Table 1:** Age, plasma NTproCNP and annualised growth velocity (AGV) at screening, and change (delta) at 6 months after commencing therapy.

	Age (years)	NTproCNP (pmol/L)	NTproCNP SDS	AGV (cm/year)	AGV SDS	Delta AGV @ 6 months (cm/year)	AGV SDS @ 6 months
Cohort 1	8.0 ± 0.6	36.2 ± 2.3	0.1 ± 0.3	3.6 ± 0.5	− 2.5 ± 0.4	0.0 ± 0.6	− 2.3 ± 0.4
Cohort 2	8.4 ± 1.0	45.4 ± 4.0	1.1 ± 0.4	3.2 ± 0.2	− 2.6 ± 0.6	0.5 ± 0.4	− 2.0 ± 0.4
Cohort 3	8.6 ± 0.6	43.5 ± 3.2	1.0 ± 0.3	4.0 ± 0.9	− 1.5 ± 0.8	2.1 ± 0.8	0.1 ± 0.4
Cohort 4	7.4 ± 0.3	43.6 ± 2.9	0.9 ± 0.3	4.2 ± 0.7	− 2.1 ± 0.7	2.5 ± 0.9	0.6 ± 0.4

### Sudden change in growth velocity is associated with a suppression of NTproCNP

**Figure 1:**
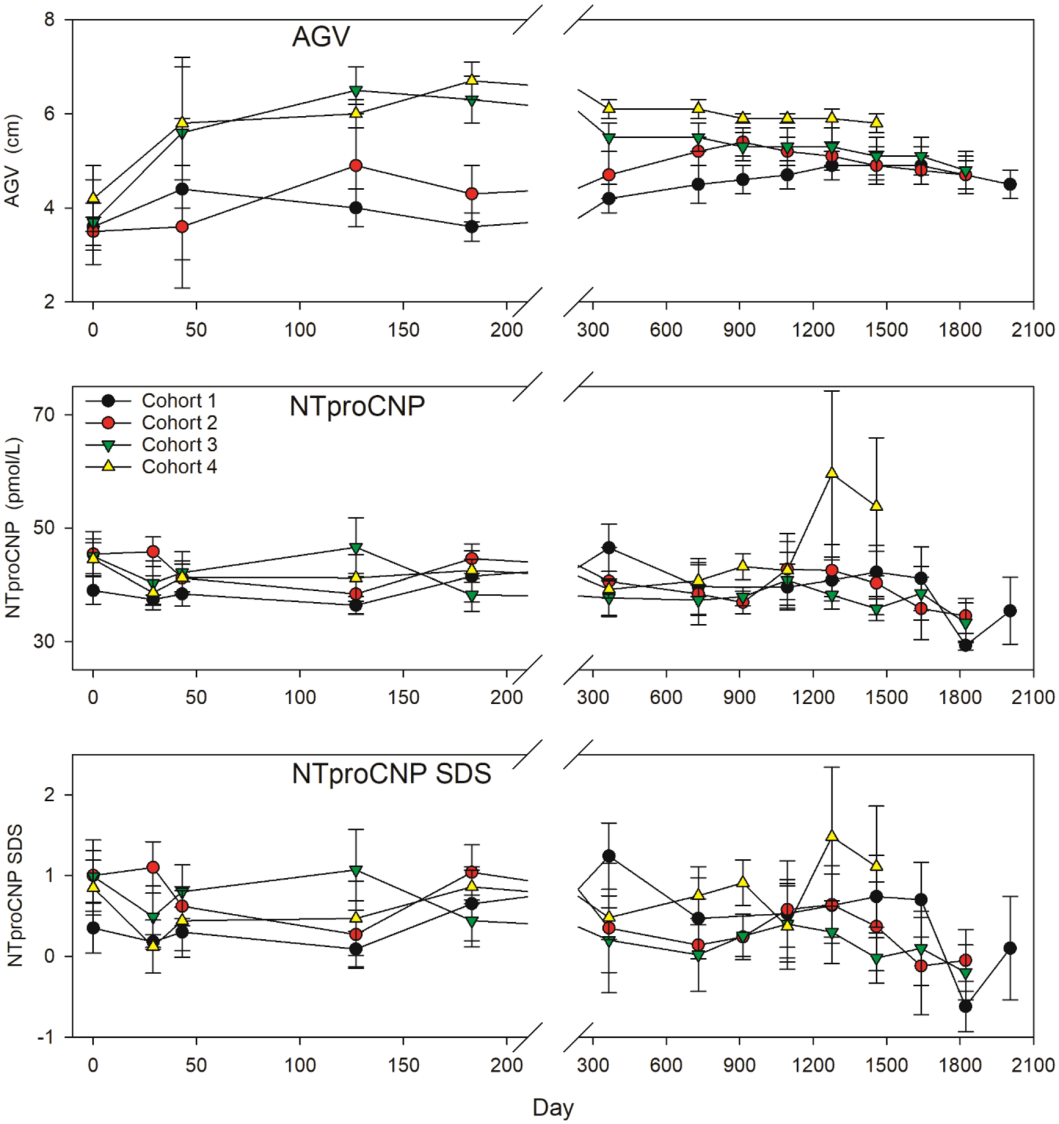
Annualised growth velocity (AGV), plasma NTproCNP concentrations and NTproCNP SDS (adjusted for age and sex) by cohort across the study. Values are mean ± SE. Cohort 1 (6 subjects, age range 6–10 year at screening) received 2.5 µg/kg/day for up to 10 months (~ to day 300), followed by 7.5 µg/kg/day for approximately 2 months (~ to day 360), and thereafter 15 µg/kg/day until study completion. Cohort 2 (6 subjects, age range 5–10) received 7.5 µg/kg/day for the initial 6–8 months (180–240 days)—escalating to 15 µg/kg/day thereafter. Cohorts 3 (8 subjects, age range 6–11) and Cohort 4 (8 subjects, age range 5–8) received 15 µg/kg/day and 30 µg/kg/day respectively throughout the study. Expected average AGV in normal children and in untreated Ach is 6 and 4 cm/year respectively^13,27^.

Dynamic changes in NTproCNP and SDS in relation to growth promoting actions of Vosoritide are shown for each cohort in Fig. 1. Changes in individuals by cohort are shown in Supplementary Date (Supplementary Fig. S1). Sharp inflexions in AGV in Cohorts 3 and 4 across the initial 3 months were associated with significant fall in NTproCNP SDS at 1 month (P = 0.04 and 0.004, cohort 3 and 4 respectively). At later time points in the first year of treatment of these two groups when AGV had stabilized, mean NTproCNP SDS was more variable but trended lower than baseline (Fig. 1). When the dose was escalated to 15 µg/kg/day after 1 year in cohorts 1 and 2, increases in AGV were associated with decline in NTproCNP (Fig. 1), but lack of frequent sampling early in the course of dose escalation prevents a more detailed analysis of temporal changes.

### Anomalous increases in NTproCNP

**Figure 2:**
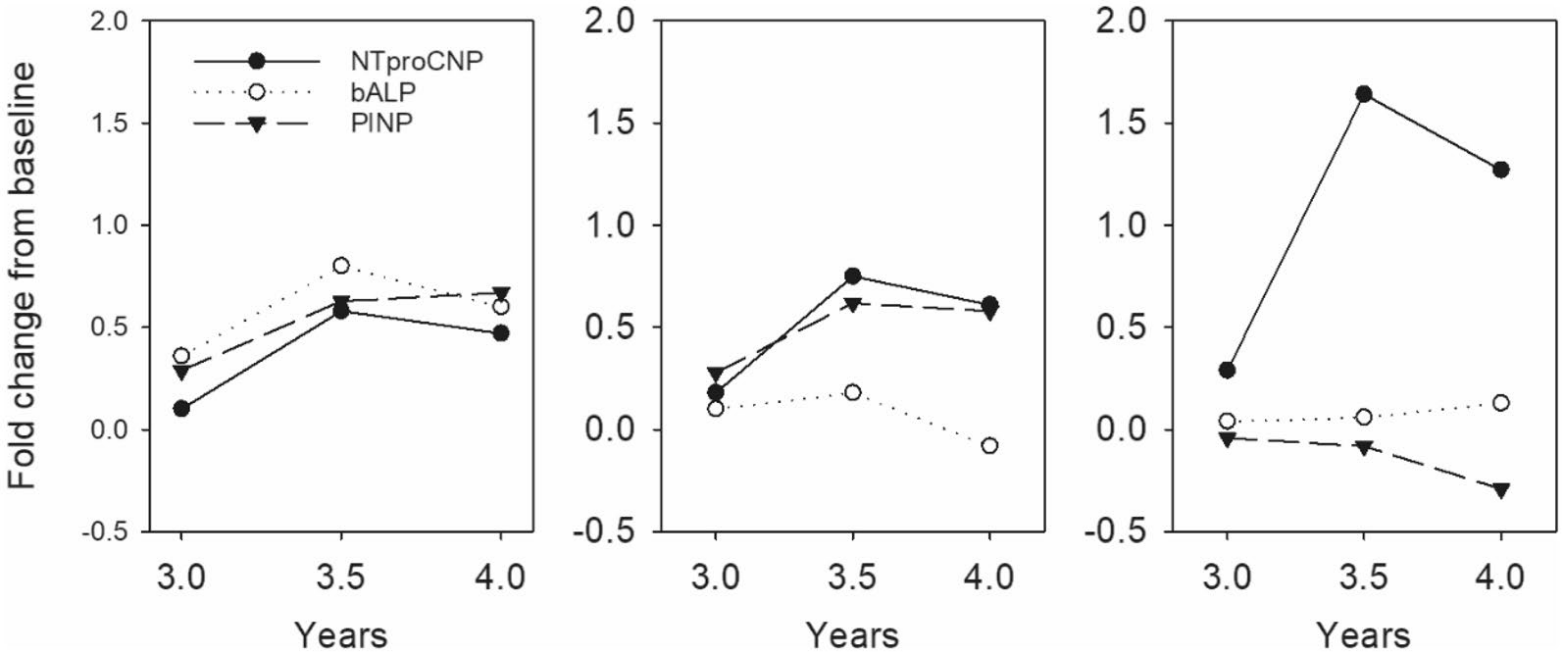
Fold change from baseline (screening) in bone turnover markers (bALP, PINP) and plasma NTproCNP in three Cohort 4 subjects in years 3–4 of therapy. Each panel depicts concurrent analyte concentrations in a single subject.

Inspection of Fig. 1 shows relatively unchanging AGV from years 2–4 associated with unchanging NTproCNP excepting those in Cohorts 4. In Cohorts 2 and 3 (both receiving 15 µg/kg dose) mean NTproCNP SDS was lower than at screening at all-time points. In Cohorts 1 and 4, several anomalous and unexplained elevations were observed—all unassociated with change in AGV. Marked increases in plasma levels were observed after 3–4 years of treatment with 30 µg/kg/day (Cohort 4) in three of eight children (Supplementary Fig. S1). Increases in SDS in these children (3.1, 6.7; 1.4, 3.0; and 0.3, 1.9—pre and peak respectively) were sustained for at least 1 year and were not consistently associated with changes in either BALP or PINP (Fig. 2). In two subjects, Tanner stage 2 pubertal development was documented but in the subject with the highest NTproCNP (138 pmol/L, SDS 6.7) puberty had not developed until breast budding was notated 6 months after the last measurement. Occasional spikes in plasma NTproCNP were observed in other subjects, some of which coincided with puberty staging (Supplementary Fig. S1). In contrast, in one female child (Cohort 1) age 6.5 year, plasma level increased abruptly some 6 months after starting the 2.5 µg/kg dose (Supplementary Fig. S1). SDS (0.16 at screening) rose to 2.6 at 6 months and remained elevated for the remaining 4.5 year but was unaffected by normal pubertal progression at age 10.5 year. No consistent link was apparent with BALP, PINP or AGV.

### Persistent growth under exogenous CNP effect was associated with persistent suppression of NTproCNP

To assess any impact of chronological age or duration of therapy on plasma NTproCNP, comparison was made between SDS at 1 year and at the study’s completion. Since Cohorts 1–3 were all receiving 15 µg/kg/day dose after 1 year, values were combined. As shown in Table 2, significant decline in NTproCNP SDS was observed over time (F = 14, P = 0.002) during periods of relatively unchanging AGV (Fig. 1).

**Table 2:** Effect of 15 µg/kg/day Vosoritide on mean NTproCNP SDS after 1–5 years of therapy (n = 17).

Study year	NTproCNP SDS
1.0	0.54 ± 0.28
4.0	0.32 ± 0.26
4.5	0.22 ± 0.29
5.0	− 0.20 ± 0.21*

*Significant difference from year 1 (P = 0.015).

### Acute exposure to exogenous CNP analogue inhibits NTproCNP production

**Figure 3:**
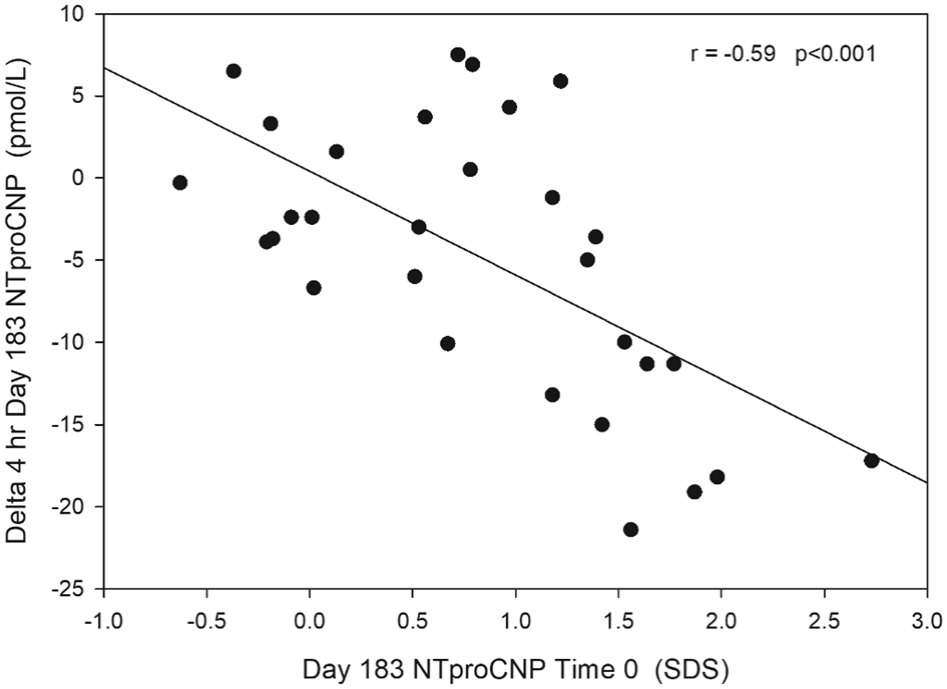
Relationship between change (delta) in plasma NTproCNP concentration at 4 h after injection on Day 183 and NTproCNP SDS prior to injection on the same day.

The impact of the first injection of Vosoritide was studied only in Cohorts 1 and 2. In Cohort 1, no change in NTproCNP 4 h after injection was observed after 2.5 µg/kg/day—nor in any of the six studies undertaken in the subsequent 10 months of treatment. In Cohort 2, significant fall (pre 43.3 ± 3.5, post 35 ± 2.7 pmol/L, P = 0.013, n = 8) resulted from the initial 7.5 µg/k dose, returning to pre injection levels (44.3 ± 4.7 pmol/L) at 8 h post injection. In these same subjects, significant suppression was observed 1 month after commencing treatment (P = 0.02) at which time AGV had not changed (Table 1). Across all subjects, results from paired samples were available on up to eight different time points during the initial 24 months of Vosoritide treatment. Since responses did not differ according to dose injected (F = 0.8, P = 0.5), results on any given time point were combined for statistical analysis. As shown in Table 3, when the grouped data was analysed no significant change at 4 h was found within the first 6 months of therapy (that is on days 29, 43 and 127). However, at each subsequent time point, a significant fall in plasma NTproCNP was observed on days 183 (P = 0.003, n = 30), 12 months (P = 0.006, n = 22) and at 24 months (P = 0.015, n = 17, see Table 3). Irrespective of significance in the decline at 4 h itself, when links with concurrent NTproCNP SDS at the time of injection were examined, associations of fall in plasma NTproCNP (delta) with pre injection plasma NTproCNP SDS were identified at screening (r =  − 0.71, P < 0.001); at Day 29 (r =  − 0.45, P = 0.012); at Day 127 (r =  − 0.42, P = 0.02); at Day 183 (r =  − 0.59, P < 0.001, see Fig. 3) and at 24 months (r = − 0.36, P = 0.10). Higher pre-test SDS strongly associates with the fall in plasma NTproCNP at 4 h. Together these results support a direct inhibitory effect of exogenous CNP on CNP production which is not observed during the initial 6 month period if skeletal growth accelerates.

**Table 3:** Change (delta) in plasma NTproCNP (pmol/L) at 4 h post Vosoritide in relation to duration of therapy.

Day	N*	Pre	Post	Delta	P value
29	28	39.9 ± 1.5	37.5 ± 1.4	− 2.4 ± 1.5	0.12
43	25	40.6 ± 1.5	40.1 ± 1.6	− 0.5 ± 1.0	0.62
127	27	41.5 ± 2.0	39.8 ± 1.8	− 1.7 ± 1.5	0.27
183	28	42.4 ± 1.4	37.3 ± 1.3	− 5.0 ± 1.6	**0.003**
365	22	40.5 ± 2.2	36.2 ± 2.2	− 4.3 ± 1.4	**0.006**
730	17	39.6 ± 2.4	35.5 ± 2.4	− 4.2 ± 1.5	**0.015**

*Number of subjects.

Significant values are given in bold.

## Discussion

The possibility that CNP production may be subject to negative feedback regulation is an important issue that needs study in light of the peptide’s increasing use in growth disorders in children. Already there is strong circumstantial evidence from genetic disorders of CNP signalling in humans for such a servomechanism, though its mechanism—direct or indirect—is quite unclear. The first administration of a CNP analogue in children with Ach has provided the opportunity to explore the dynamic response of endogenous CNP secretion, as measured by concentrations of NTproCNP in plasma, to exogenous doses of Vosoritide over an extended period of 5 years. Although constrained by the small sample size, variations in dose regimens, timing and frequency of sampling, and mindful that the dynamics of CNP regulation in Ach may differ in other settings, the study reveals several key observations. First, NTproCNP SDS at baseline is significantly increased. Second, significant declines in plasma NTproCNP during the initial acceleration in AGV by growth-promoting doses of Vosoritide provides evidence of feedback regulation, which is likely to be indirect. Third, the decline in plasma NTproCNP 4 h after injection of Vosoritide—in proportion to plasma NTproCNP SDS just prior to injection—is consistent with a direct feedback effect. These and other findings of marked increase in NTproCNP in early adolescence in some subjects receiving higher doses are important new observations that call for closer study.

Studies from rodent pups, growing lambs and children support the view that circulating levels of CNP products in plasma (CNP and NTproCNP) are sourced largely from growth plate or closely related tissues^4^. Measurement of any possible impact on endogenous proCNP production of administered CNP 1–37 pro gly (which strongly cross-reacts with CNP but not in the NTproCNP assay) is therefore only feasible using plasma concentrations of NTproCNP adjusted for age and sex (SDS). As previously reported^6^ we confirm levels in healthy children with Ach are significantly increased prior to treatment—and remain so until the final phase of the study. Notably, during doses that sharply increased AGV within 2–3 months (15 and 30 µg/kg/day in Cohorts 3 and 4), NTproCNP SDS fell significantly, coinciding with the first signs of increments in serum collagen X marker, a degradation product of type X collagen^13^. These changes were not observed in subjects receiving lower doses (2.5 and 7.5 µg/kg/day) where AGV was unaffected at this time. It is instructive to compare these dynamic changes in CNP with those found in short non Ach children of similar age starting daily doses of Human Growth Hormone (HGH)^15^. In that setting, similar inflections in AGV were associated with *increase* in NTproCNP (mean 11 pmol/L, delta 22% at day 21) in contrast to the concurrent *fall* of NTproCNP (approximately 6 pmol/L, range 3–11 pmol/L, delta − 12%) at Day 29 during 15–30 µg/kg/day Vosoritide treatment in Cohorts 3 and 4. Disparate responses are unsurprising since growth plate concentrations of CNP products are clearly increased by growth hormone but not by exogenous CNP. These kinetic responses could relate to the estimated times (approximately 20–22 days)^16,17^ for recruited chondrocytes to traverse the respective zones of the expanding growth plate and populate the primary spongiosa. However, unlike the persisting elevation in NTproCNP above baseline during the first year of HGH treatment^15^, the initial marked depressions in concentrations during Vosoritide therapy were less sustained—possibly reflecting the much shorter half-life of Vosoritide (28 min) compared to the much longer duration of HGH activity (up to 12 h)^15^. Taken together, the current findings are consistent with an indirect feedback mechanism as postulated in other genetic disorders^7–9^, whereby a factor generated by accelerated growth plate activity—or osteoid tissue—reduces local *NPPC* expression or secretion of proCNP into the extra cellular fluid. They also align with the indirect negative feedback from exogenous CNP observed in 4 week old rodent pups^12^. However in that study, 3 days of continuous high dose intra venous infusions of CNP 53, while significantly reducing lumbar vertebral *Nppc* expression, did not reduce plasma NTproCNP in male rats (n = 7) and were associated with a small decline in females (n = 6). No indices of skeletal or growth plate activity in this brief exposure to CNP 53 were reported so possible links of accelerating endochondral bone growth with reduced plasma NTproCNP in this setting remain to be studied. Further study of larger groups of subjects and more appropriately timed sampling points, particularly within the initial 3 months of starting exogenous CNP therapy, can be expected to advance understanding of these dynamic changes in relation to changing bone growth in children, and may provide clinical applications. For example, decline in NTproCNP at 1 month—or targeting zero NTproCNP SDS in Ach—could be used to predict optimal effect size, duration of effect on growth plate activity and choice of dose and frequency of injections.

An unexpected finding that remains largely unexplained is the marked increase in NTproCNP SDS in some subjects, most evident in early adolescence in those receiving the highest dose. In at least three subjects, this increase was sustained for 1 year, and in one other child the increase commenced at age 6 years and was sustained for at least 4 years. Since serial sampling of CNP products in untreated children with Ach has not been studied, it is possible that these large variations are intrinsic and linked to the underlying tissue resistance in this disorder. In most of these excursions (which were not sex dependent), there was no consistent association of NTproCNP with bone turnover markers, which is not surprising in light of their different time course in response to changes in bone turnover in adults^18^. Renal function (as reflected by serum creatinine), known to strongly affect plasma NTproCNP, was normal in all cases and did not change. No accurate monitoring of pubertal timing or staging was possible in this exploratory multi-centre study so absence of any consistent relationship with pubertal development^19^ does not exclude synergistic effects of sex steroids on osteogenesis which may involve increased expression of CNP^20,21^. Periosteal apposition during adolescence also needs to be considered in light of the reported increase in CNP gene expression during differentiation of cells of periosteal lineage in tissue culture^22^. Significant decline in NTproCNP SDS towards zero after 4 years of 15 µg/kg/day treatment (when AGV was unchanging) possibly reflects time dependent (indirect) feedback effect of Vosoritide in osteoid tissue as the earlier and positive skeletal impacts of puberty begin to wane. Study of these patterns was not possible in those receiving the highest dose (Cohort 4) as these subjects were significantly younger and the duration of serial CNP sampling significantly shorter (see Fig. 1). Further study is required to dissect out possible associations of plasma CNP products—not only during adolescence in untreated Ach but also the impact of CNP analogues on CNP secretion associated with pubertal timing and bone maturation.

As detailed above our first hypothesis—that feedback action of Vosoritide would be indirect—was supported by our data. However, our second hypothesis—that CNP would not be subject to acute (direct) feedback—was not. Surprisingly, in multiple studies undertaken at different times across the initial 2 years of treatment strong evidence for acute feedback was revealed but only after 6 months of exogenous therapy. This temporal constraint may relate to the early growth spurt observed in many of children studied. Those in cohorts 3 and 4 exhibited initial sharp inflections in AGV-reducing plasma NTproCNP drawn 24 h post injection in this period-which may limit any decrease at 4 h from an injected dose. Neither group was tested prior to one month but no suppressive impact from either 15 or 30 µg dose was seen on day 29, 43, 127 or day 183 (when AVG was accelerating) but was clearly observed at later time points. AGV in the first 6 months was not significantly affected in either cohort 1 or cohort 2. In the former, the initial dose was insufficient to affect bioactivity (urine cGMP) on day 1^13^, or consistently increase plasma CNP 39 within 2 h^13^, and did not affect AGV. Lack of suppression at 4 h in any of the 8 studies conducted is therefore unsurprising. On the other hand, after 7.5 µg/kg/day (cohort 2) significant increase in urine cGMP and peak CNP 39 was observed on day 1 along with significant suppression of NTproCNP at 4 h—also observed in this cohort on day 29. Unfortunately, during the period of dose escalation in these 2 cohorts, sampling frequency was insufficient to assess any impact of accelerating AGV on NTproCNP- or the possible effect of this on the response at 4 h. While other factors such as increase in body weight, puberty, dose and bioavailability (much greater in cohort 3 and 4)^13^ and the small number of subjects studied need to be considered, collectively the findings suggest that an interaction between indirect and direct feedback systems may account for the reduced impact of Vosoritide at 4 h in the first 6 months. Combining all groups, highly significant decline in NTproCNP at 4 h after dosing on days 183, 365 and 730 was found (Table 3). Notably, evaluating the response across all studies according to the NTproCNP SDS shows that the acute fall (effect size) is strongly dependent on the SDS at the time of dosing. This finding suggests that by restoring intra cellular CNP activity via a functional receptor (NPR2), CNP production is reduced in proportion to the prevailing level of resistance. Conceivably increased expression of the clearance receptor NPR3, reducing CNP and increasing NTproCNP (the antithesis of loss of function in NPR3^8^) could account for the findings we observe. However, since both CNP and NTproCNP are similarly increased in untreated children with Ach^6^ and the ratio NTproCNP/CNP is normal, upregulation of NPR3 is unlikely. Of note, no direct feedback was found in wild type rodent pups^12^—raising the possibility that our findings of suppression (which were not sex dependent) could be specific to Ach where circulating CNP products are elevated above normal. In clinical (safety) studies (BMN 111–101) undertaken in healthy normal adult males, no significant change in plasma NTproCNP from baseline was observed at 4 h after doses ranging from 2.5 to 15 µg/kg/day. This suggests that direct suppression characterises the immature skeleton but whether confined to Ach requires further study—for example in children without the genetic disorder—as well as in Ach where the SDS is close to zero. Notwithstanding these findings, it is unlikely that the direct feedback we observe contributes to CNP regulation in vivo considering the very high concentrations (> 350 pmol/L peak)^13^ associated with significant suppression, and levels seen in pathophysiology (2–8 pmol/L)^6^.

Summarising these feedback actions of exogenous CNP, we propose that *indirect* feedback (assessed here by the plasma concentration of NTproCNP 24 h post injection) involves a delayed—dose dependent—response of expanding growth plate tissues analogous to the well described feedback loop of paracrine signals secreted by hypertrophic cells on stem cell recruitment from the resting zone^23^. We suggest that this indirect feedback is the basis of the strong inverse relationships between intracellular CNP activity and circulating CNP products in genetic disorders affecting CNP signalling. On the other hand, *direct* feedback is evident at 4 h—consistent with the temporal dynamics linking cGMP inhibition of FGFR3^24^—is lost at 8 h, and is dependent on the prevailing degree of intracellular resistance to CNP activity at the time of study. Except for the intersection of the two feedback systems soon after long bone growth is initiated, direct inhibition is unrelated to AGV. The underlying molecular mediators of these events remain to be clarified.

In conclusion, we show that endogenous CNP is still subject to regulation during daily administration of growth promoting doses of the CNP analogue Vosoritide. In keeping with the restraining impact of the genetic mutation on CNP signalling, baseline levels of CNP products in plasma are raised. During periods of accelerating long bone growth induced by Vosoritide, CNP is reduced in keeping with an indirect negative feedback. Direct (acute) inhibition of CNP by exogenous CNP occurs in proportion to the prevailing level of hormone resistance. Unusually high values of plasma NTproCNP in adolescent years in some subjects with Ach subjects receiving Vosoritide is unexplained and requires further study.

## Materials and methods

### Subjects and study design

Subjects, study design (Study 901; 202; 205: NCT 01603095; NCT 02055157; NCT 02724228 respectively), safety and efficacy of Vosoritide in Ach have been recently published^13^. In brief, 35 children (5–14 years of age) were enrolled in four sequential cohorts at nine study sites in this dose—finding and extension study. Because measurements of plasma NTproCNP were available only in 28 of these subjects (age range 5–11, 12 male, 16 female), all data presented here apply to this subgroup alone. After screening at baseline, four separate cohorts—balanced by sex but distinguished by dose and timing of dose escalation—received daily subcutaneous injections of Vosoritide for periods up to 5.5 years. Cohort 1 (6 subjects, age range 6–10 year at screening) received 2.5 µg/kg/day for up to 10 months, followed by 7.5 µg/kg/day for approximately 2 months, and thereafter 15 µg/kg/day until study completion. Cohort 2 (6 subjects, age range 5–10) received 7.5 µg/kg/day for the initial 6–8 months—escalating to 15 µg/kg/day thereafter. Cohorts 3 (8 subjects, age range 6–11) and Cohort 4 (8 subjects, age range 5–8) received 15 µg/kg/day and 30 µg/kg/day respectively throughout the study. At completion of NTproCNP sampling, respective ages (means) were 13.2 year in Cohort 1, 13.7 year in Cohort 2, 13.6 year in Cohort 3 and 11.4 year in Cohort 4. Signs of pubertal development were observed after 2 year of treatment in all except in three subjects in Cohort 2, one subject in Cohort 3, and in two subjects in Cohort 4. Except on days requiring blood sampling, Vosoritide was injected subcutaneously by trained caregivers at the subject’s home. All studies were performed in accordance with the provisions of the Declaration of Helsinki, and all study procedures were approved by the relevant ethics boards at each site: Vanderbilt HRPP, Nashville, TN, USA; Institutional Review Board for Baylor College of Medicine, TX, USA; UCSF Benioff Children’s Hospital Oakland IRB, Oakland, CA, USA; The Royal Children’s Hospital Melbourne Human Research and Ethics Committee, Victoria, Australia; Ann & Robert H. Lurie Children's Hospital of Chicago Institutional Review Board, IL, USA; Comite de Protection des Personnes Ile de France VIII, Boulogne-Billancourt, France; Johns Hopkins Medical Institution Review Board, Baltimore, USA; John F Wolf Human Subjects Committee (2), Torrance, CA, USA; East Midlands—Nottingham 2 Research Ethics Committee, Nottingham, England. Informed consent was obtained from all subjects and/or their legal guardian(s).

### Laboratory tests and procedures

#### Plasma NTproCNP

To assess possible correlations with changes in annualised growth velocity (AGV) and or during puberty, serial (pre injection) sampling was done throughout the study. EDTA anticoagulated plasma was collected at screening (baseline), then at 12 different time points during the 5 year period. Anticipating links between endogenous CNP and the initial phase of growth acceleration^15^, sampling was more frequent in all cohorts during the initial 2 months of therapy. Measurements were also made on day 85 in Cohort 1, in three subjects in Cohort 2 but not other cohorts, so are not included in the analysis of serial changes in AGV. Sampling was less frequent after 1 year (at 1, at 2 year and then half yearly until final sampling at year 5.5).

To examine possible acute effects of Vosoritide on plasma NTproCNP, samples were drawn 4 h after injection in all subjects on nine separate occasions across the first two years of the study. In Cohort 1 and 2 subjects, samples were drawn at both 4 h and 8 h after the first injection. All other samples were drawn 4 h after the routine morning injection.

All plasma NTproCNP measurements were carried out in duplicate by the Christchurch laboratory assay using their previously determined age and sex adjusted normal ranges (Standard Deviation Scores, SDS) for normal children^19^. There was no detectable cross-reactivity of CNP (1-37pro gly) in the NTproCNP assay. This assay has a detection limit of 1.5 pmol/L, and within and between assay coefficients of variation of 6 and 7% respectively at 18 pmol/L. In previous studies of 6 healthy young adults (unpublished) values showed no evidence of diurnal fluctuation or effect of food ingestion during 0900 h and 1500 h (average coefficient of variation 6.4%)—and no significant variation in samples drawn at short intervals on successive days. Because both age and sex affect plasma concentrations of NTproCNP in normal children^25^ all measured concentrations of NTproCNP were converted to SDS using data from reference ranges previously determined from 258 normal healthy children aged 2 months to 20 year^19^. As SDS for plasma NTproCNP are not available for *untreated* Ach, the above approach was deemed to be the optimal comparator for use in these samples drawn from children of differing age and sex, because the tempo and pubertal timing of height velocity in untreated Ach does not seem to differ from those in normal non Ach children^26^.

#### Other relevant measurements

AGV (cm per year) was calculated from measurements of standing height at intervals of 3 months. AGV measured during the last 6 months before screening was used for baseline values.

Pubertal grading employed Tanner stage 1 to 5 using breast development and pubic hair in girls, and testis size and pubic hair in boys. Staging was recorded at each visit after 1 year of treatment.

Bone turnover markers (bone alkaline phosphatase, BALP and the aminoterminal procollagen propeptide of type 1 collagen, PINP) were measured as part of routine lab procedures pre injection and in most cases concurrently with NTproCNP sampling.

### Statistics

Results are expressed as mean ± S.E. Comparison of NTproCNP SDS data with those of the general population (mean: 0, SD: 1) were made using one-sample Student’s t-test. Comparisons between the cohort groups were done using a two-tailed Student’s t-test or repeated measures ANOVA where appropriate. Where significant changes were observed with analysis of variance, Bonferroni post hoc analysis was used to detect differences from baseline values or timed interval data as appropriate. Spearman’s rank coefficient was used to determine correlations between variables, presented as r values. Statistical significance was assumed when P < 0.05.

## Supplementary Information

Supplementary Figure S1.

## Data Availability

Some or all datasets generated during and/or analyzed during the current study are not publicly available but are available from the corresponding author on reasonable request.
