# Supplementary material for: Evidence of feedback regulation of C-type natriuretic peptide during Vosoritide therapy in Achondroplasia
Source: Sci Rep. 2021 Dec 20;11:24278. doi: 10.1038/s41598-021-03593-1 (PMC8688426; doi:10.1038/s41598-021-03593-1)
Supplement: Supplementary file 1 — Supplementary Figure S1. [file 41598_2021_3593_MOESM1_ESM.docx]

**Supplementary Information**

**Evidence of feedback regulation of C-type Natriuretic Peptide during Vosoritide therapy in Achondroplasia.**

Timothy CR Prickett^1*^, Eric A Espiner^1^, Melita Irving^2^, Carlos Bacino^3^, John A Phillips III^4^, Ravi Savarirayan^5^, Jonathan RS Day^6^, Elena Fisheleva^6^, Kevin Larimore^6^, Ming Liang Chan^6^, George S. Jeha^6^.

^1^ Department of Medicine, University of Otago, Christchurch, New Zealand

^2^ Guy's and St. Thomas' NHS Foundation Trust, Evelina Children's Hospital, London, UK

^3^ Baylor College of Medicine, Houston, TX, USA

^4^ Vanderbilt University Medical Center, Nashville, TN, USA

^5^ Murdoch Children’s Research Institute, Royal Children’s Hospital Victoria, University of Melbourne, Parkville, Victoria, Australia

^6^ BioMarin Pharmaceutical, Novato, California, United States of America.


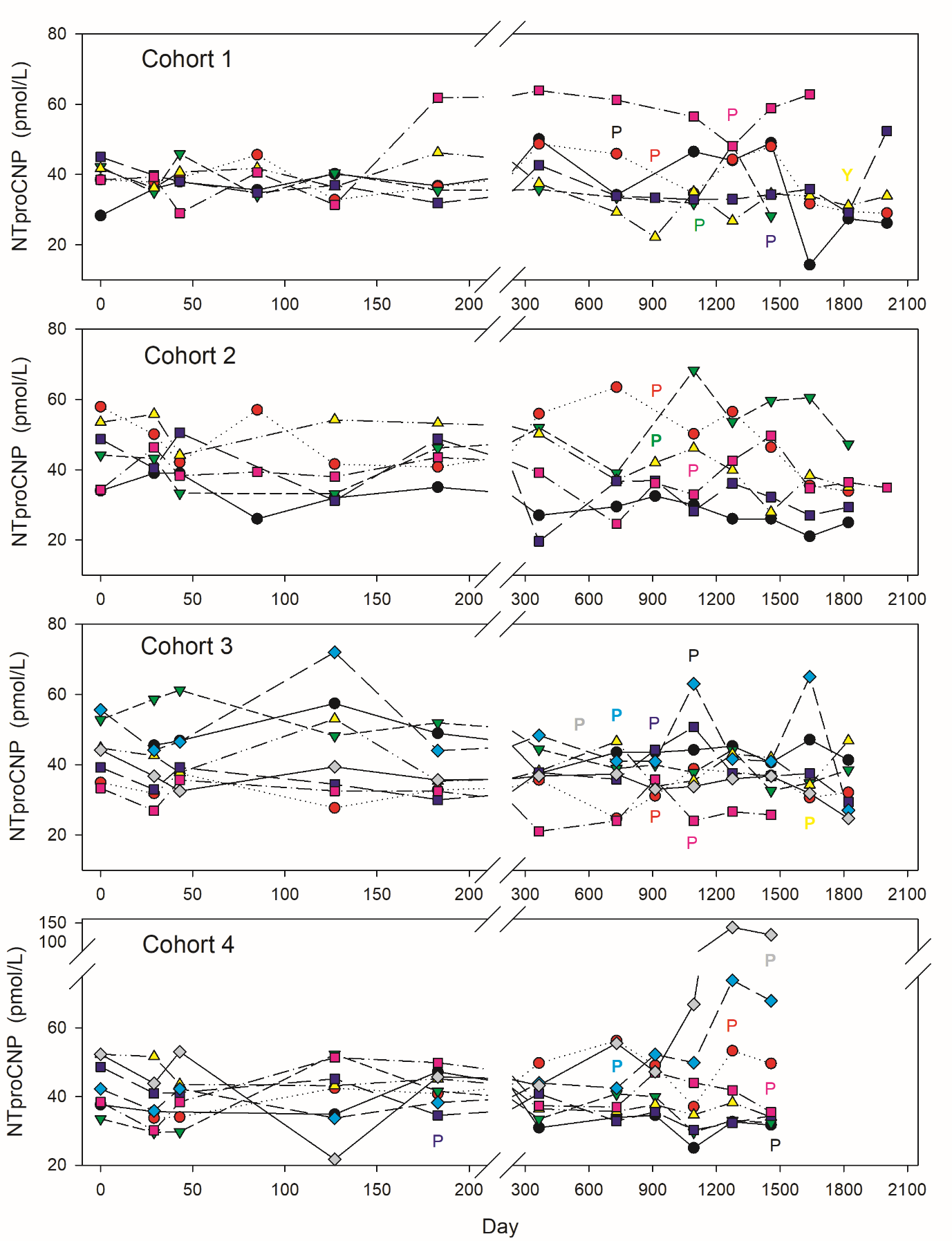


**Supplementary Figure S1.** Changes in NTproCNP concentration over time by cohort. Individuals within each cohort are delineated by colour. The letter P denotes the time of the visit when the individual was determined to have reached Tanner stage 2. Cohort 1 (6 subjects, age range 6-10yr at screening) received 2.5 µg/kg/d for up to 10 months (~ to day 300), followed by 7.5 µg/kg/d for approximately 2 months (~ to day 360), and thereafter 15 µg/kg/d until study completion. Cohort 2 (6 subjects, age range 5-10) received 7.5 µg/kg/d for the initial 6-8 months (180-240 days) – escalating to 15 µg/kg/d thereafter. Cohorts 3 (8 subjects, age range 6-11) and Cohort 4 (8 subjects, age range 5-8) received 15µg/kg/d and 30µg/kg/d respectively throughout the study.
